# Floppy Closing Door Epiglottis Treated Successfully with an Mhealth Application Based on Myofunctional Therapy: A Case Report

**DOI:** 10.1155/2019/4157898

**Published:** 2019-07-01

**Authors:** Carlos O'Connor Reina, Guillermo Plaza Mayor, Jose Maria Ignacio-Garcia, Peter Baptista Jardin, Maria Teresa Garcia-Iriarte, Juan Carlos Casado-Morente

**Affiliations:** ^1^Chief of Department of Otorhinolaryngology, Hospital Quiron Salud Marbella, Hospital Quiron Salud Campo de Gibraltar, Cádiz, Spain; ^2^Chief of Department of Otorhinolaryngology, Hospital Sanitas La Zarzuela, Hospital Universitario Fuenlabrada, Fuenlabrada, Spain; ^3^Chief of Neumology Department, Hospital Quiron Salud Marbella, Hospital Quiron Salud Campo de Gibraltar, Cádiz, Spain; ^4^Otorhinolaryngology Department, Clinica Universitaria de Navarra, Navarra, Spain; ^5^Otorhinolaryngology Department, Hospital La Merced, Osuna, Sevilla, Spain

## Abstract

We introduce the first case reported to date of a floppy closing door epiglottis in an OSA (obstructive sleep apnea) patient treated successfully with an Mhealth smartphone application based on myofunctional therapy.

## 1. Introduction

Myofunctional therapy (Mt) has become one of the latest treatments used for sleep-disordered breathing [1]. It is based on the periodical use of daily exercises using oropharyngeal muscles to try to strengthen them, thereby helping to open the upper airway. Obstructive sleep apnea (OSA) originates from the lack of optimal functioning of the dilator muscles of the airway. Therefore, Mt aims theoretically at treating the pathophysiology underlying this disease [2]. Mt is based on oropharyngeal exercises described by diagrams and videos instructed by a myofunctional therapist, usually on a weekly basis. Patients have to perform these exercises for at least 3 months, between 20 and 40 minutes daily. However, in most cases, patients perform exercises by themselves at home without proper feedback and without giving exact information to the consultant about the performance of the exercises [3].

AirwayGym® is a novel Mobile-Health (MHealth) application (App) designed to promote oropharyngeal exercises interacting with a smartphone in those patients who cannot afford a myofunctional therapist or do not tolerate other therapeutic options, such as continuous airway pressure (CPAP) support and mandibular advancement devices (MADs), or do not want to undergo upper airway surgery.

**Figure 1:**
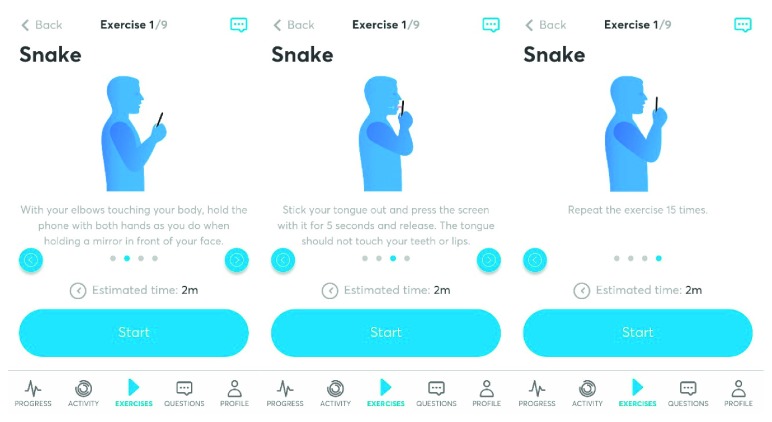
Exercises.

**Figure 2 fig2:**
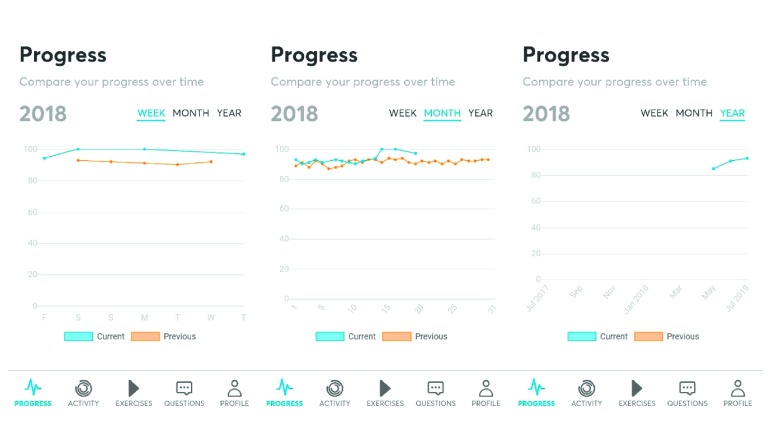
Evolution.

**Figure 3:**
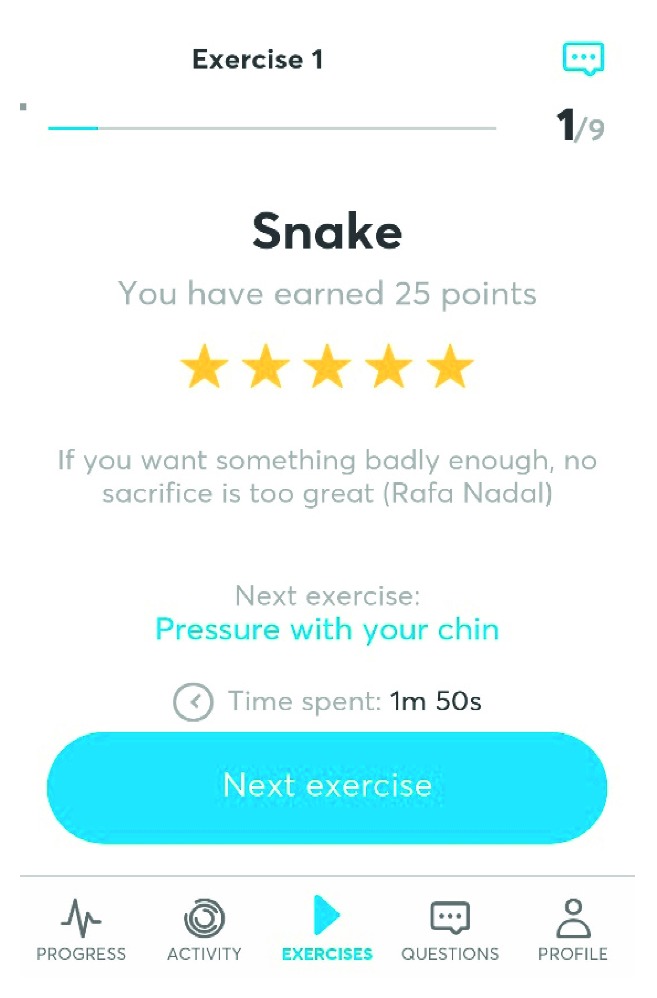
Score.

The Airway Gym® application has been developed with the most up-to-date technologies (Ionic and Angular provided by Google and TypeScript by Microsoft) and using the most consolidated coding (such as HTML5, CSS3, and PHP). Furthermore, it takes advantage of cutting-edge technology in the devices that have it installed as a three-dimensional touch facility. Such a capacity is ready for use in the latest Apple and Android devices to accurately measure the pressure that is produced on the smartphone screen. By using this App, patients have a permanent record of feedback about the accuracy of their exercises carried out when interacting with the screen. Exercises are saved in the Cloud, and a faculty trainer can control the progress of activity by contacting each patient using an online chat facility. The English version of Airway Gym® is presently offered on Android and iOS platforms via Google Play and Apple Store (Figures 1 [Fig fig2]–3).

Airway Gym offers a series of daily exercises to do with the smartphone. Patients can perform them at any time simply by interacting with the smartphone's screen. In total, there are nine exercises to be performed daily taking 15–20 min. These exercises are isometric and isotonic to improve the tone and coordination of the dilator muscles of the airway. So far, most people who have performed the exercises correctly every day for 3 months have significantly improved their snoring and apnea problems [3].

The App includes the following:

1. Exercises: the main objective of the App
2. Activity: getting to know each patient's daily activity in detail
3. Progress: personal trainers can monitor each patient's progress over time with graphs
4. Queries: patients can directly consult their personal trainer using a private chat facility while maintaining confidentiality of their personal contact numbers
5. Notifications: if patients wish, they can receive notification on the smartphone to start the exercises at a preferred time
6. Help: there is a form within the App by which every patient can send their suggestions or questions

Floppy closing door epiglottis, or trapdoor epiglottis, is a collapsible epiglottis that blocks the airway and is one of the most challenging situations that a sleep surgeon can encounter [4]. Its diagnosis is based on performing drug-induced sleep endoscopy (DISE) [5]. This technique allows the surgeon to identify the different levels of obstruction causing OSA. Up to now, the only effective treatment of a floppy epiglottis has been surgery to remove the epiglottis totally or partially with laser, coblator, or robotic surgery [4]. Nevertheless, surgery involves serious risks such as permanent bronchoaspiration, inspiratory dyspnea, and swallowing problems. Therefore, Mt might be a safe treatment option for patients with a floppy epiglottis.

## 2. Case Presentation

A 50-year-old man came to our Ear, Nose, and Throat (ENT) Department with OSA that was intolerant to the CPAP and MAD treatments recommended by his pneumologist. He complained of progressive severe somnolence and headaches. Between his antecedent treatments, he had had two heart infarcts and underwent cardiac artery bypass surgery 3 years ago. He was under anticoagulant medication. He also suffered from high blood pressure controlled with calcium-channel inhibitors.

The ENT examination showed no anatomical findings that suggested the reason for his OSA. We observed a Friedman stage 1, with tonsil size grade 1. An examination of his nose showed no obstruction, and the size of the base of the tongue was normal. His body mass index was 22.1 kg/m^2^. His Epworth sleepiness scale was 22. In a sleep study performed using polygraphy, his apnea-hypopnea index (AHI) was 31.2 and minimal O_2_ saturation was 81.3%.

As part of our ENT protocol, DISE is generally indicated when a patient does not tolerate any therapeutic option and a surgical solution might be needed. Following the European consensus guidelines, DISE was performed under an anesthetist's control using propofol. In this case, DISE showed a floppy closing door epiglottis (video in Supplementary materials (available here)) and it was diagnosed as the cause of the collapse during apneas. During DISE, there were no modifications of the collapse when changing the patient's head position to lateral or after an Esmarch maneuver, excluding a positional reason.

Three expert sleep surgeons evaluated the video and considered that the only valuable option was a partial epiglottoplasty under general anesthesia. For personal reasons, the patient rejected such surgery. We then offered Mt, but he could not afford it. After obtaining his consent, we then offer him the use of the Airway Gym® App for 90 sessions and arranged for periodical follow-ups.

The patient noticed that his headache and somnolence were reduced gradually. We then arranged a new DISE (video in Supplementary materials (available here)) after 3 months; surprisingly, the floppy closing door epiglottis had disappeared. The three expert sleep surgeons agreed that surgery was not now necessary. We arranged a new sleep study in which the AHI was reduced to 17.2, minimal O_2_ saturation improved to 85.1%, and his Epworth sleepiness scale improved to 15. In this new situation, a MAD was recommended but rejected by the patient for economic reasons.

## 3. Discussion

This is the first case reported where Mt performed by an Mhealth App helped avoid mandatory surgery. As there were no significant changes in the normal lifestyle of our patient, we assume that the improvement seen was only achieved using the Airway Gym® App. Once the floppy closing door epiglottis was resolved this patient was more comfortable with the other therapeutic options available. Now, this patient continues to use this App every day because he noticed progressive improvements with its use. We decided to submit this case report because of the high prevalence of people who have no adherence to their treatments to control their OSA [6].

OSA is a chronic disease and will be the most prevalent breathing disease in the next few years. Only one out of four patients is diagnosed, and only half of those receive proper treatment [7]. Thus, proper diagnoses and treatments will save plenty of healthcare resources. Telemedicine has the option to be a major tool for treating this disease. The universality of the Android and iOS platforms gives sleep doctors a major option for facing the problems of connectivity, feedback, and adherence that affect these patients and drive them to abandon their treatments.

Our App has been introduced in several meetings [3, 7, 8] and now there are more than 1,200 users worldwide. We are performing a multicentric clinical trial with the Code AWAG-2019-01, and results will be available ending this year. In our patient, after 15 months of using the App, his AHI levels remained reduced and steady and an MAD had been rejected because of its price.

Being the first case reported of a floppy epiglottis apparently cured using an Mt-based smartphone App, we do not have a clear scientific reason to explain our finding. We understand that Mt increases tone [1] in the muscles of the tongue, also achieving a reduction in its fat content.

These changes are supposed to “tense” the glossoepiglottic folds, and this tension might act like suspenders that prevent the collapse of the epiglottis. Further studies are needed to support this suggestion.

This is a small sample of what clinicians can achieve with MHealth technology using smartphones. In this case, Mt performed with a patient using an App has avoided a hitherto “unavoidable” surgery. We understand that this is only one case but, given our experience, we think that in the future there will be plenty of such cases that will help to improve our standard of care and open our minds about other options for treating sleep disorders.

## Supplementary Materials

Supplementary Materials DISE showing changes of a floppy trap door epiglottis after 90 sessions with an App based on myofunctional therapy.
